# Community Factors and County-Level Cancer Screening, Prevalence, and Mortality

**DOI:** 10.1001/jamanetworkopen.2025.37690

**Published:** 2025-10-23

**Authors:** Alexandra R. Drake, Eric W. Christensen, Augusto C. Ochoa, William Small, Jinel Scott, Elizabeth Y. Rula

**Affiliations:** 1 Harvey L. Neiman Health Policy Institute, Reston, Virginia; 2 Department of Interdisciplinary Oncology, Louisiana State University Health Sciences Center, New Orleans; 3 Department of Radiation Oncology, Stritch School of Medicine, Cardinal Bernadin Cancer Center, Loyola University Chicago, Maywood, Illinois; 4 Department of Radiology, State University of New York Downstate Health Sciences University, Brooklyn; 5 Office of Quality and Safety, New York City Health and Hospitals/Kings, Brooklyn

## Abstract

**Importance:**

Cancer screening, prevalence, and mortality vary across US counties; the broad set of community factors associated with these differences are not well understood.

**Objective:**

To assess the relative importance of diverse community measures for explaining county-level variance in cancer screening, prevalence, and mortality rates for breast, colorectal, lung, and prostate cancers.

**Design, Setting, and Participants:**

This geospatial cross-sectional analysis across all US counties used random forest algorithms to estimate the relative importance of 24 community measures—including health behaviors and lifestyle, socioeconomic, and environment factors—as explanatory factors for cancer screening, prevalence, and mortality. For each cancer, county-level cancer screening and prevalence rates were based on a nationally representative 5% sample of Medicare fee-for-service beneficiaries (2020). The 5-year (2016-2020) mean mortality rates were from the National Cancer Institute’s State Cancer Profiles. Neiman Cancer disparity maps geospatially display significant community-factor to cancer-outcome associations via county-level choropleth maps. Data were analyzed from September 2024 through February 2025.

**Exposure:**

Residence in the 50 states, Puerto Rico, and the District of Columbia.

**Main Outcomes and Measures:**

Outcomes were county-level rates of cancer screening, prevalence, and mortality for breast, colorectal, lung, and prostate cancers. Separate random forest algorithms estimated the relative importance of 24 community measures for each outcome–cancer type combination, and maps display county-level associations.

**Results:**

Among a nationally representative 5% of 2020 Medicare Fee-For-Service beneficiaries, 87% were aged 65 years or older. The top ranking explanatory community factors (100% relative importance) for mortality were smoking rate for both lung and colorectal cancers, and the non-Hispanic Black population share for both breast and prostate cancers. For prevalence and screening rates, the top factors were unique for each cancer type. Uninsured rate, unemployment, limited access to health foods, and poor physical health were the top ranked factors for breast, colorectal, lung, and prostate cancer prevalence, respectively. Hispanic population, poverty, air pollution, and Air Toxics Cancer Risk were ranked highest for screening rates, respectively, for each cancer. Two of only 3 environmental factors, Environmental Justice Index and Air Toxics Cancer Risk, had the most top-5 associations for mortality and screening, respectively. Uninsured rates for prevalence and poverty rates for screening were also important across cancer types.

**Conclusions and Relevance:**

In this cross-sectional study of the relative explanatory importance of community measures on cancer screening, prevalence, and mortality rate we found that the ranking of factors varied for each specific cancer and outcome, which can inform targeted population health efforts. Factors that ranked high across cancer types for a given outcome, such as uninsured rates for cancer prevalence, represent opportune targets for future study and broader policy change.

## Key Points

**Question:**

What is the relative importance across a diverse set of community factors for explaining county-level differences in screening, prevalence, and mortality rates for breast, lung, prostate, and colon cancers?

**Findings:**

In this cross-sectional study including all US counties, random forest modeling found that the relative explanatory importance of community measures on cancer screening, prevalence, and mortality rates differed, both within and across cancers. Geospatial patterns also varied, revealing regional and county-level needs.

**Meaning:**

These findings suggest that the relative importance of community factors and specific local and regional needs together can inform prioritization and direct resources to the most opportune focus areas to impact cancer outcomes.

## Teaser

This cross-sectional study evaluates the association of a diverse set of community factors with county-level variance in screening, prevalence, and mortality rates for breast, colorectal, lung, and prostate cancers.

## Introduction

Over 2 million new cancer cases will be diagnosed in the US and there will be over 600 000 deaths, according to 2024 estimates.^1^ Approximately 40.5% of people will be diagnosed with cancer during their lifetime.^1^ In addition to morbidity and mortality, cancer also accounts for a significant portion of total US health care spending. The costs of cancer-related health care could total $246 billion by 2030, 34% higher than 2015 costs. Forty-three percent of that cost was borne by Medicare (33%) and Medicaid (10%), while more was carried by private insurers (49%).^2^ In 2019, the economic burden of cancer care to patients was $21.09 billion, including the highest out-of-pocket medical costs for breast ($3.14 billion), prostate ($2.26 billion), colorectal ($1.46 billion), and lung ($1.35 billion) cancers.^3^

However, cancer risk is not evenly distributed across the population or geographical regions. While genetics play a role, a range of external factors contribute to the risk to individuals and populations. There is growing recognition that the inclusion of community-level social risk measures improves the performance of models that predict health care use, which are increasingly used to direct services and policy. The County Health Rankings model, an evidence-based model for how community characteristics impact health, estimates that 80% to 90% of what impacts health is not medical care, but socioeconomic status (40%-50%), health behaviors and lifestyle (30%-40%), and environmental factors (5%-10%).^4,5,6,7,8,9^ Various studies show the association of health behaviors and lifestyle^10,11,12,13,14,15^ and socioeconomic status^11,12,15,16,17^ with health or mortality. Income^18,19,20^ and education^15,20,21,22^ have shown consistent associations with health. While studies differ on the exact magnitude of the impact, the association is evident.

Given the importance of community-level factors on population health, we sought to evaluate the relative importance of these factors in relation to prevalent cancers, given that cancer is the second leading cause of death, only slightly behind heart disease.^23,24^ Cancer deaths are often preventable. For example, policy changes that decreased smoking and increased cancer screening have significantly reduced cancer mortality.^25^ With the aim of providing actionable information to reduce the impact of cancer, we focused on the screening, prevalence, and mortality rates for 4 cancer types—breast, prostate, lung, and colorectal—that account for 50% of new cancer cases.^26^ In 2024, the Harvey L. Neiman Health Policy Institute published interactive cancer disparity maps.^27^ At the US county level, these maps allow the user to examine the association of cancer prevalence, screening, and mortality rates with various community measures. However, the associations displayed in the maps do not indicate which factors are most important.

A previous study assessed the relative importance of various factors with all-cancer mortality at the US county level using a random forest (RF) model. However, to our knowledge, the importance of a broad set of community factors on a range of cancer outcomes for specific, prevalent cancers has not been quantitatively examined. Also using an RF model, our study assesses the relative importance of 24 community measures (in the categories of health behaviors and lifestyle and health, socioeconomics, environment, race and ethnicity, and health care access) with each combination of cancer outcome (screening, prevalence, and mortality) and cancer type (breast, prostate, lung, and colorectal). We hypothesize that the relative importance of these community measures will vary by outcome–cancer type combination. Better understanding of the relative importance of various community measures on these outcomes can better inform, prioritize, and target efforts to improve cancer outcomes and lead to prospective research to reveal causal relationships and test interventions.

## Methods

### Data Sources and Study Population

This retrospective study was deemed exempt from oversight by the Advarra institutional review board. The study was conducted in 2024, following Strengthening the Reporting of Observational Studies in Epidemiology (STROBE) reporting guidelines. County-level screening and prevalence rates were calculated for breast, colorectal, lung, and prostate cancer from a nationally representative 5% of 2020 Medicare Fee-For-Service beneficiaries. Eligibility rules for rate calculations were developed using screening recommendations from the American Cancer Society for each cancer type (breast: female patients aged 40-80 years; colorectal: both sexes aged 45-85 years; lung: both sexes aged 50-80 years; prostate: male patients aged 50-80 years).^28,29,30,31,32,33^ Counties with fewer than 11 eligible beneficiaries were excluded from the analysis and map creation, as is standard in Centers for Medicare and Medicaid Services data aggregation. County-level, age-adjusted, 5-year mean mortality rates for breast, colorectal, lung, and prostate cancer were obtained from the National Cancer Institute’s state cancer profiles for the period of 2016 to 2020. County-level community factors were obtained from 3 sources: Area Health Resource File,^34^ County Health Rankings and Roadmaps,^4^ and the Environmental Justice Index (EJI) provided by the Agency for Toxic Substances and Disease Registry for the 2020 calendar year.

### Outcomes and Community Measures

The outcome measures include cancer screening, prevalence, and mortality rates for breast, prostate, lung, and colorectal cancers. Community measures were collected at the county level. The county-level community factors (eTable in Supplement 1) included population demographics and 18 measures in the 4 categories of the County Health Rankings model: 6 health behavior and lifestyle and health factor rates (smoking, excessive drinking, insufficient sleep, physical inactivity, obesity, and poor physical health); 7 socioeconomic factors (rates of poverty, unemployment, education, access to exercise opportunities, severe housing problems, limited access to healthy foods, and residential segregation index); 3 environment factors (air pollution, air toxics cancer risk, EJI); 6 race and ethnicity factors, assessed because this variable is associated with different cancer outcomes (percentage of Asian, Hawaiian, Hispanic, non-Hispanic Black, non-Hispanic White, and North American Native from self-identified race collected by the US Census Bureau); and 2 health care access measures (percentage of uninsured adults and primary care physicians per 100 000 population). Note that other community measures, such as income, food insecurity, other race, environmental burden, and air quality index, were considered but excluded due to overlap or collinearity with other measures, determined by a correlation coefficient greater than 0.7.

### Statistical Analysis

To determine the relative importance of the various county-level community measures with cancer screening, prevalence, and mortality rates for breast, prostate, lung, and colorectal cancers, we used RF algorithms. RFs have been shown to explain more variation in the data as measured by R-squared than linear regression because they are not constrained by the linear assumptions or specific functional forms.^35^ However, unlike linear regression for which the linear assumption and specific functional form yields easy interpretation, RFs are machine-learning models that do not provide any information about how model predictions were made.^35^

Instead, RFs use the relative importance of the model variables to provide insight into the importance of each variable. The relative importance score for the most important variable in the model is set to 100%. All other variables are then normalized relative to the most important variable. A variable that is one-half as important as the most important variable has a relative importance of 50%. Given each variable’s relative importance, the results can be used to assess the degree to which a specific variable may be highly important for one outcome but not another. Note that the model estimates do not indicate the direction of the correlation measure with the outcome variable, but only the strength of the association relative to the other measures. We conducted RFs for each of the 12 combinations of outcomes (screening, prevalence, and mortality) and cancer type (breast, prostate, lung, and colorectal), and optimized the models to minimize error in the training process and the depth of the decision tree. Analyses were conducted in Stata 18 (StataCorp) from September 2024 through February 2025.

## Results

**Figure 1.**
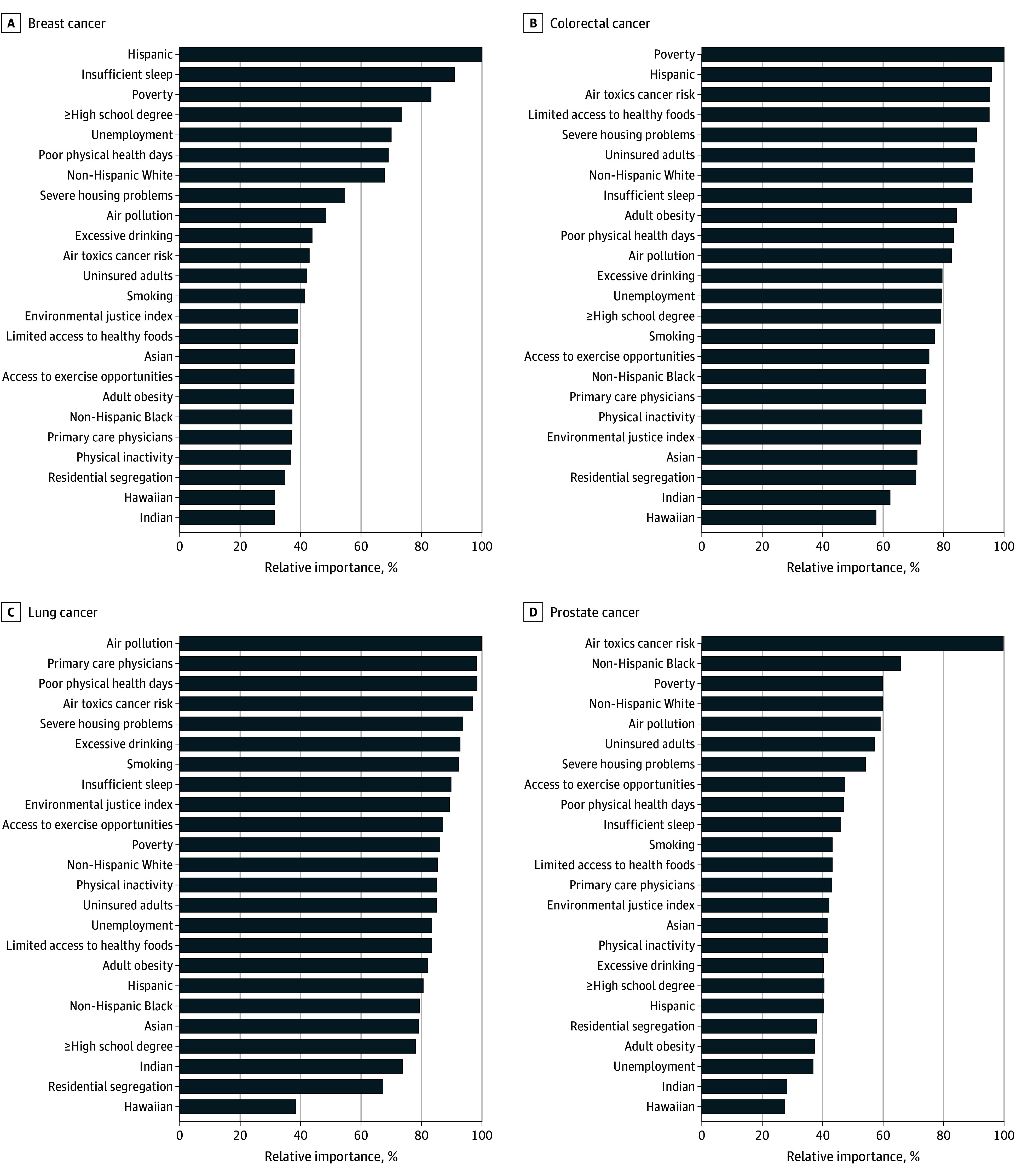
Relative Importance of Community Measures for Cancer Screening by Cancer Type

**Figure 2.**
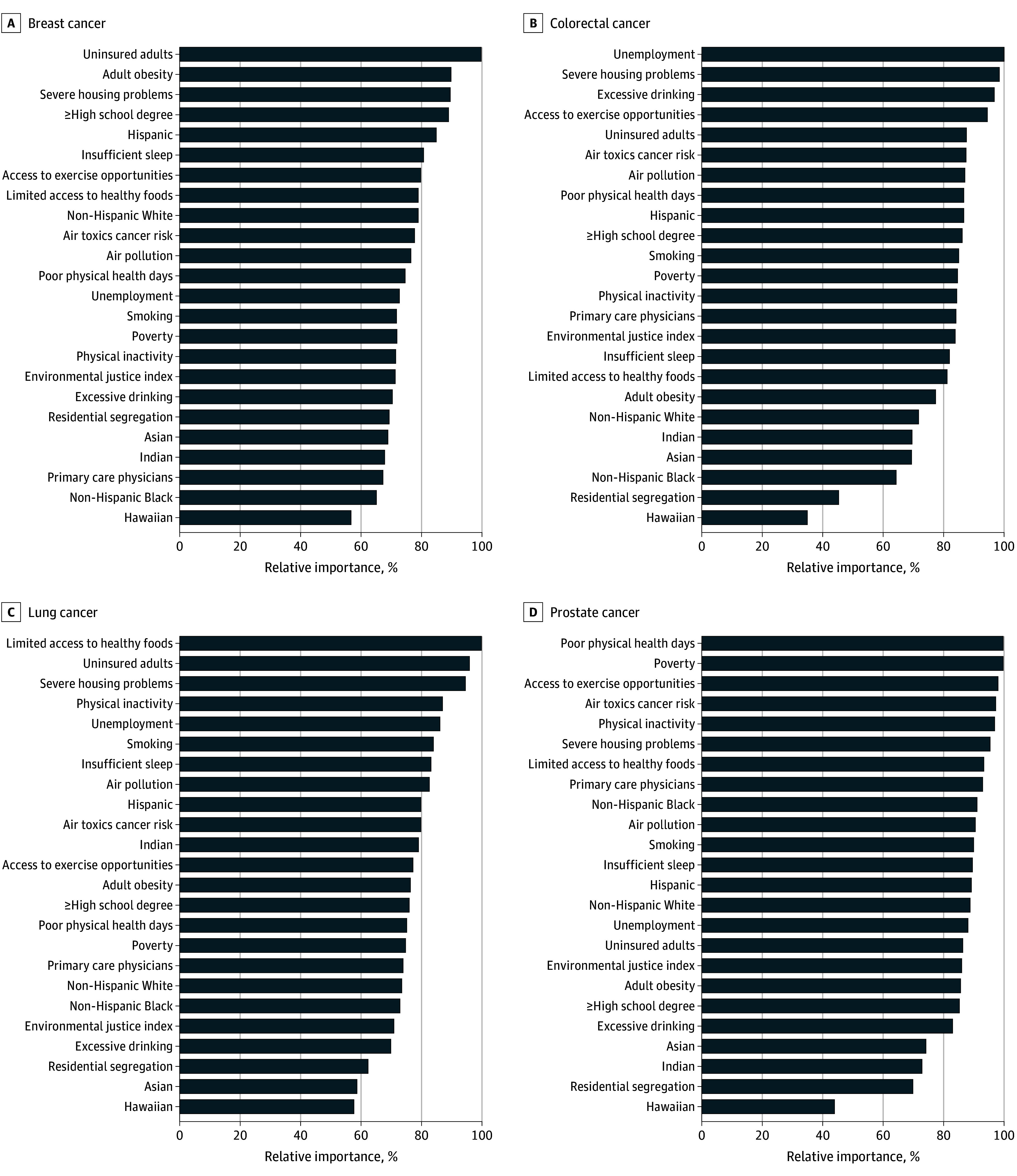
Relative Importance of Community Measures for Cancer Prevalence by Cancer Type

**Figure 3.**
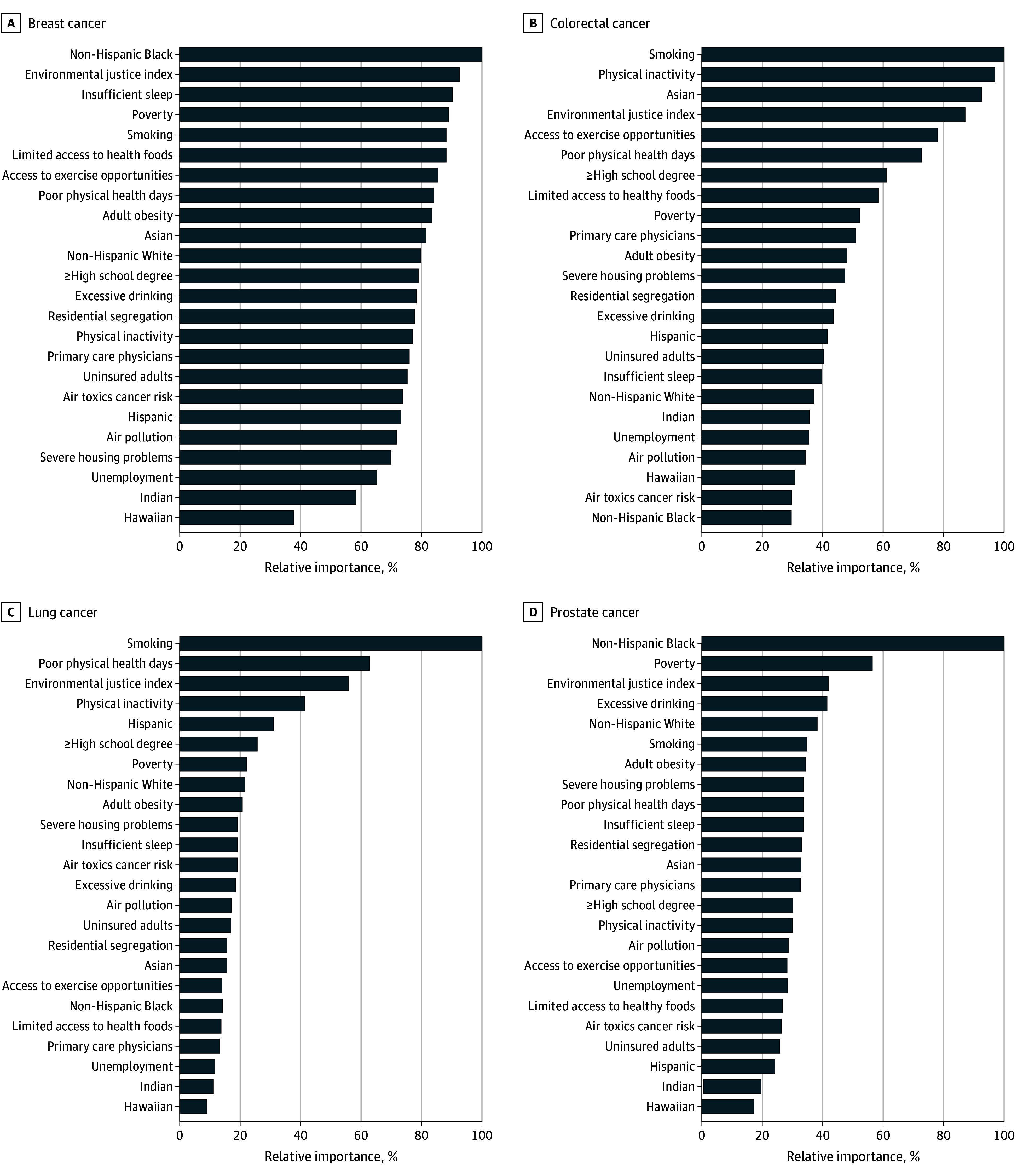
Relative Importance of Community Measures for Cancer Mortality by Cancer Type

Among a nationally representative 5% of 2020 Medicare Fee-For-Service beneficiaries, 87% were aged 65 years or older. The relative importance of the 24 community measures by cancer type is shown for cancer screening (Figure 1), prevalence (Figure 2), and mortality (Figure 3). For example, the Hispanic population share was the most important factor for breast cancer screening, while uninsured rates and the non-Hispanic Black population share were the most important factors for breast cancer prevalence and mortality, respectively. Similarly, the relative importance of community measures differed by cancer type. For lung cancer, air pollution and access to primary care physicians were the most important factors for screening, limited access to healthy foods and uninsured rates were the most important for prevalence, and smoking was by far the most important for mortality. Poverty, unemployment, and smoking were the most important factors for colorectal cancer screening, prevalence, and mortality, respectively. For prostate cancer, the most important factors were air toxics cancer risk (screening), poor physical health days (prevalence), and the non-Hispanic Black population share (mortality).

We found that for some outcome–cancer type combinations, there was substantial variance in the relative importance across the various community measures (large gradient), whereas for other combinations the measures contributed similarly in relative importance. For example, relative importance of the measures differed little for lung cancer screening and prevalence for breast, lung, and prostate cancers. In contrast, the gradient was large for screening of breast and prostate cancers and mortality of colorectal, lung, and prostate cancers. Specifically, for breast cancer prevalence, the eleventh ranked factor (air pollution) had a relative importance of 76.5%, but for prostate cancer mortality, the second (poverty) and third (EJI) had relative importances of 56.5% and 42.1%, respectively. Hence, considering both the rank and relative importance percentage are necessary in examining the results.

**Figure 4.**
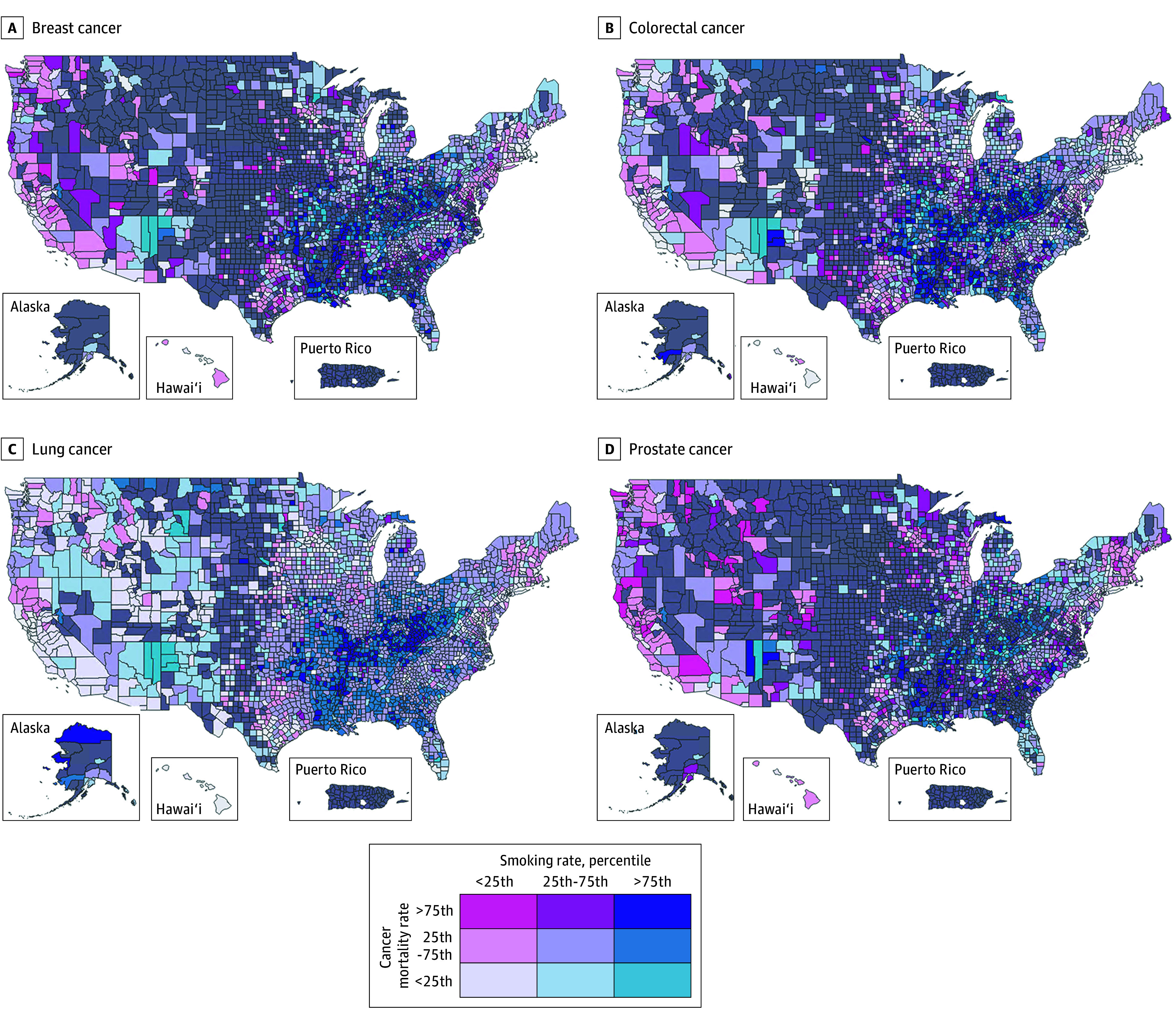
Cancer Disparity Maps Showing Cancer Mortality and Its Association With Smoking, by County and Cancer Type Key indicates color scheme by quartile of each variable (middle quartiles combined into 25th-75th percentile range); The top right section of the key (indigo) indicates least favorable combination; the bottom left (pale lavender) the most favorable. Gray indicates missing data or small sample.

Among the health behaviors and lifestyle measures, smoking was the most important for mortality for colorectal and lung cancer and ranked fifth in importance for breast cancer. The association between mortality and smoking is shown in the cancer disparity maps (Figure 4), which show higher smoking and mortality in the South and lower smoking and mortality in the West and Northeast. Smoking rate was in the top 3 most important measures for the prevalence of lung and prostate cancer. Smoking rate was relatively less important for screening than it was for prevalence and mortality across all cancers. Insufficient sleep ranked among the top 3 most important measures for breast cancer screening and mortality.

**Figure 5.**
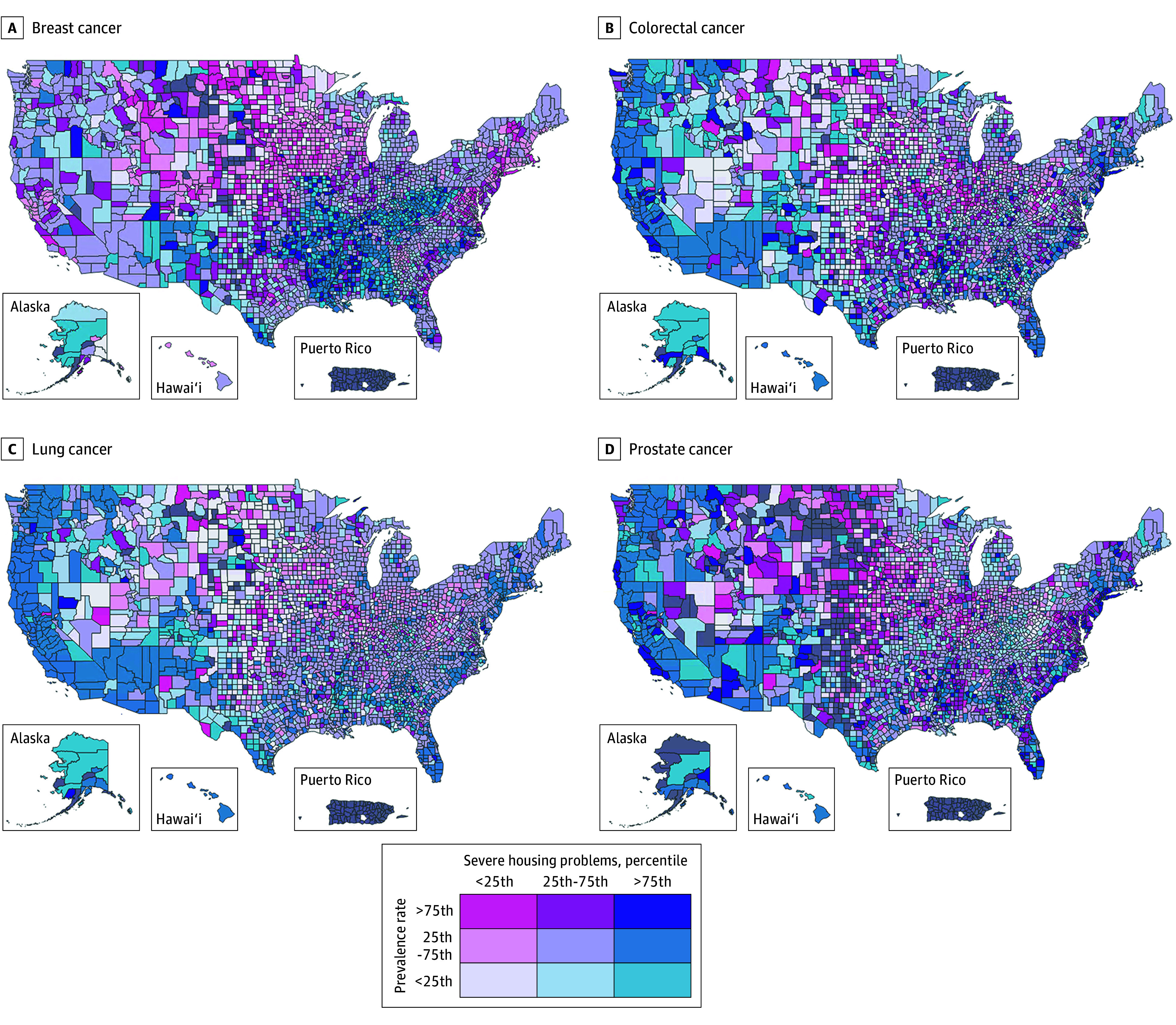
Cancer Disparity Maps Showing Cancer Prevalence and Its Association With Severe Housing Problems, by County and Cancer Type Key indicates color scheme by quartile of each variable (middle quartiles combined into 25th-75th percentile range); The top right section of the key (indigo) indicates least favorable combination; the bottom left (pale lavender) the most favorable. Gray indicates missing data or small sample.

Among the socioeconomic measures, severe housing problems emerged as higher importance in prevalence models, particularly for breast, colorectal, and lung cancer (Figure 2), and ranked sixth or eighth in all screening models (Figure 1). Cancer disparity maps demonstrate a regional association between severe housing problems and cancer prevalence that was greatest in the West and along the East coast, except for breast cancer, where the strongest correlations were in the mid-South (Figure 5). Poverty had high relative importance for colorectal, breast, and prostate cancer screening and was among the top 3 risks for all prostate cancer models. The cancer disparity maps show poverty’s association with cancer screening (eFigure in Supplement 1), with higher poverty levels associated with lower screening rates from the 4 corners area along the southern border to the Gulf Coast as well as in the South, particularly for lung and breast cancer screening.

Among environmental measures, air toxics cancer risk emerged as highest in relative importance for prostate cancer screening. For lung cancer screening, air pollution and the air toxics cancer risk ranked first and fourth, respectively. For cancer mortality, the EJI ranked between second and fourth for all cancer types, but was lower in rank for cancer screening and prevalence models.

Among the race and ethnicity measures, the Hispanic population share was the most important measure for breast cancer screening and second for colorectal cancer screening. The non-Hispanic Black population share ranked second for prostate cancer screening and had the highest relative importance for both breast and prostate cancer mortality. The Asian population share ranked third in importance for colorectal cancer mortality. Health care access measures generally had lower relative importance than other categories of community measures. Neither of the 2 health care access measures showed high relative importance broadly across the outcomes or cancer types. The exceptions were that uninsured adults were among the top 5 measures for breast, lung, and prostate cancer prevalence.

## Discussion

This study analyzed the relative importance of county-level community factors associated with screening, prevalence, and mortality by cancer type. Prior research has established the important role of an individual’s community on health^5,6,8^ and proposed causal molecular pathways for this effect in cancer.^36^ Various health behaviors and lifestyle, socioeconomic, and race and ethnicity measures demonstrated high relative importance across all cancers and outcomes, with relatively lower importance for environmental factors and health care access measures. The importance of individual measures varied, however, with no measure(s) consistently ranking at the top for all cancer types studied. Hence, individual measure importance must be considered uniquely for each outcome–cancer type combination. Our results are consistent with the literature demonstrating the importance of health behaviors and lifestyle, socioeconomic status, and environmental factors on population health.^5,6,7,8,9^

Various studies show the association of health and mortality with health behaviors and lifestyle.^10,11,12,13,14,15^ We found that health behaviors and lifestyle measures had high relative importance for cancer mortality and prevalence. Smoking had the greatest relative importance in association with colorectal and lung cancer mortality, as expected, as well as a high relative importance for breast cancer mortality, ranking in the top 5 measures. Prior research has shown smoking to be a cause of cancer and associated with all-cancer mortality^37,38^ and breast cancer mortality rates.^39^ Although tobacco control efforts have significantly reduced smoking prevalence, it still remains the leading cause of lung cancer and related mortality,^40^ and our results show that smoking rate was less important for screening rates than the other cancer measures, indicating a need to focus on cancer screening in these areas.

We found that insufficient sleep had a relative importance higher than 80% for cancer screening (breast, colorectal, and lung), prevalence (all 4 cancers), and breast cancer mortality. A possible mechanism for this association is provided by research demonstrating that melatonin, which regulates circadian rhythm, plays a role in preventing tumor development.^41,42^ Circadian rhythm disorders have been associated with breast cancer risk in some studies involving night-shift workers, but findings are inconclusive. The high relative importance of insufficient sleep and inconclusive evidence for its effect on cancer risk warrants additional studies to explore the association.

Socioeconomic status has been associated with health and mortality^9,11,12,15,17^ accounting for 40% to 50% of what impacts health.^5,6,7,8,9^ Uninsured adults, unemployment, and limited access to health food were the most important factors for breast, colorectal, and lung cancer prevalence, respectively. Severe housing problems, which include overcrowding, high housing costs, and lack of plumbing or kitchen facilities, received high relative importance in association with breast, colorectal, lung, and prostate prevalence rates. A recent review showed that overcrowded dwellings resulted in hazardous conditions and negative effects on overall health.^43^ Urinary lead levels have been associated with female breast cancer,^44^ and both elevated blood lead levels^45^ and radon exposure^46^ are associated with lung cancer risk. The prior research is consistent with our findings.

Environmental factors have been shown to be relatively less important than other community measure categories, accounting for 5% to 10% of what impacts health.^5,6,7,8,9^ We found that 1 or more environment factor ranked high in importance for cancer screening and mortality. Of the environmental measures, air toxics cancer risk and/or air pollution ranked among the top 5 for 3 screening and 1 prevalence model, but not for any of the mortality models. However, EJI, previously associated with poor health, particularly in neighborhoods with high proportions of minoritized individuals,^47^ had high relative importance for all 5 mortality models. Future research should explore the direct vs indirect effects of the environment on cancer prevention and outcomes, which may explain their importance in these models.

We found that race and ethnicity had high relative importance, particularly for cancer screening and mortality. Specific to mortality, the non-Hispanic Black population share was the most important factor for both breast and prostate cancer prevalence. American Cancer Society data show a lower breast cancer incidence in Black compared with White women, but a 40% higher mortality rate.^48^ This mortality difference has been persistent over time.^49^ Similarly, non-Hispanic Black men with prostate cancer have a 43% higher risk of death than non-Hispanic White men.^50^ With the association between EJI and poor/fair health in neighborhoods with high proportions of minoritized individuals and substantially higher breast and prostate cancer mortality among Black individuals, future studies should examine the association of EJI with race on cancer mortality.

Choropleth maps provided complementary geospatial information, demonstrating differences across the populations of US counties that reveal opportunities to improve cancer outcomes both nationally and locally. Results of the modeling reported herein can direct national priorities via the ranking of the most important factors associated with cancer outcomes nationally, and the maps provide local and regional data guide priority areas of focus for state and county policy and initiatives. Such efforts to mitigate modifiable risk factors can improve public health and also reduce the economic burden of cancer to the US economy and financial toxicity to Americans.^2,3^ Further, this work may serve as a framework that can be applied with other community factors and diseases.

### Limitations

This study has limitations. First, the analysis was conducted at the county level. Community measures may vary substantially within a county; however, the data did not support the analysis on a more granular basis due to small sample sizes. Second, relative importance is an indication of importance in predicting variation in the data, not of a relationship’s causality. Third, community measures were based on the total population or adult population. Given that this study focused on cancer screening, prevalence, and mortality, these populations may differ somewhat from the population for which cancer is more prevalent. Similarly, the generalizability of some results is limited by use of a Medicare sample to estimate cancer screening and prevalence rates, which limits generalizability to individuals who are uninsured and under age 65 years. American Cancer Society guidelines recommend screening initiation before Medicare age. Also, only age-based criteria for screening eligibility were used to estimate screening rates; data for other factors that determine individual eligibility, such as smoking history, were not available, but model estimates were adjusted for county smoking rates and demographic factors. The year 2020 was used for the analysis because it was the most recent year of time-aligned data across most measures; however, the COVID-19 pandemic may have influenced some measures, particularly screening and new diagnosis rates. Additionally, the community measures included in this study may not be representative of other measures by category (ie, the broader construct). In particular, the 2 studied health care access measures are insufficient to draw conclusions about the relationship between access and cancer metrics.

## Conclusions

This cross-sectional study detailed the relative importance of various community measures contained in the Neiman cancer disparity maps with cancer screening, prevalence, and mortality for breast, colorectal, lung, and prostate cancer. These maps visually illustrate the association of community and outcome measures and how they are regionally concentrated throughout the US. The relative importance of these measures varied across outcome measures and cancer type. Understanding the relative importance of community factors can better inform strategies to improve outcomes as well as facilitate hypothesis generation for future studies.

## Notes

Supplement 1. eTable. Description and Source for Community Measures eFigure. Cancer Disparity Maps

Supplement 2. Data Sharing Statement
