# Supplementary material for: Community Factors and County-Level Cancer Screening, Prevalence, and Mortality
Source: JAMA Netw Open. 2025 Oct 23;8(10):e2537690. doi: 10.1001/jamanetworkopen.2025.37690 (PMC12550642; doi:10.1001/jamanetworkopen.2025.37690)
Supplement: Supplement 1. — eTable. Description and Source for Community Measures eFigure. Cancer Disparity Maps [file jamanetwopen-e2537690-s001.pdf]

Supplemental Online Content

Drake AR, Christensen EW, Ochoa AC, Small W, Scott J, Rula EY. Community factors and county-level cancer screening, prevalence, and mortality. *JAMA Netw Open*. 2025;8(10):e2537690. doi:10.1001/jamanetworkopen.2025.37690

eTable. Description and Source for Community Measures

eFigure. Cancer Disparity Maps

This supplemental material has been provided by the authors to give readers additional information about their work.

© 2025 Drake AR et al. *JAMA Network Open*.

eTable. Description and Source for Community Measures

Category and measure	Description	Original Source
**Health behaviors/lifestyle and health**		
Smoking	Percentage of adults who are current smokers	Behavioral Risk Factor Surveillance System
Excessive drinking	Percentage of adults reporting binge or heavy drinking	Behavioral Risk Factor Surveillance System
Insufficient sleep	Percentage of adults who report <7 hours of sleep on average	Behavioral Risk Factor Surveillance System
Physical inactivity	Percentage of adults (aged ≥18 years) reporting no leisure-time physical activity	Behavioral Risk Factor Surveillance System
Adult obesity	Percentage of adult population (aged ≥18 years) that reports a body mass index ≥30	Behavioral Risk Factor Surveillance System
Poor Physical Health Days	Average number of physically unhealthy days reported in past 30 days (age-adjusted).	Behavioral Risk Factor Surveillance System
**Socioeconomic**		
Poverty	Percentage of population below poverty	American Community Survey, 5-year estimates
≥high school education	Percentage of adults (aged ≥25 years) who received a high school degree or higher	American Community Survey, 5-year estimates
Residential segregation	Index of dissimilarity where higher values indicate greater residential segregation between non-white and white county residents	American Community Survey, 5-year estimates
Unemployment	Percentage of population (aged ≥16 years) unemployed but seeking work	Bureau of Labor Statistics
Access to exercise opportunities	Percentage of population with adequate access to locations for physical activity	ArcGIS Business Analyst and ArcGIS Online; YMCA; US
Severe housing problems	Percentage of households with at least 1 of 4 housing problems: overcrowding, high housing costs, lack of kitchen facilities, or lack of plumbing facilities	Comprehensive Housing Affordability Strategy (CHAS) data
Limited access to healthy foods	Percentage of the population who are low-income and do not live close to a grocery store	USDA Food Environment Atlas
**Environmental**		
Air pollution	Part of the Environmental Justice Index; calculated using ozone levels, PM2.5 levels, Diesel particulate matter, and Air Toxics Cancer Risk	ATSDR, Environmental Justice Index
Air Toxics Cancer Risk	Lifetime cancer risk from inhalation of air toxics	ATSDR, Environmental Justice Index
Environmental Justice Index	Index of environmental justice that uses Social Vulnerability, Environmental Burden, and Health Vulnerability categories to calculate	ATSDR, Environmental Justice Index
**Demographic**		
Asian population	Percentage of Asian people	Census Redistricting Files
Hawaiian population	Percentage of Hawaiian people	Census Redistricting Files
Hispanic population	Percentage of Hispanic people	Census Redistricting Files
Non-Hispanic Black population	Percentage of Non-Hispanic African American people	Census Redistricting Files
Non-Hispanic White population	Percentage of Non-Hispanic White people	Census Redistricting Files
North American Native population	Percentage of North American Native people	Census Redistricting Files
**Healthcare access**		
Uninsured adults	Percentage of adults aged <65 years without health insurance	Small Area Health Insurance Estimates
Primary care physicians	Ratio of population to primary care physicians	Area Health Resource File/American Medical Association

© 2025 Drake AR et al. *JAMA Network Open*.

eFigure. Cancer Disparity Maps showing cancer screening and its association with the population share below the poverty level, by County and cancer type. Key indicates color scheme by quartile of each variable (middle quartiles combined into 25th-75th percentile range); The top right section of the key (indigo) indicates least favorable combination; the bottom left (pale lavender) the most favorable. Gray indicates missing data or small sample.

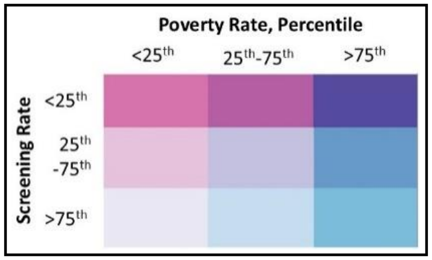

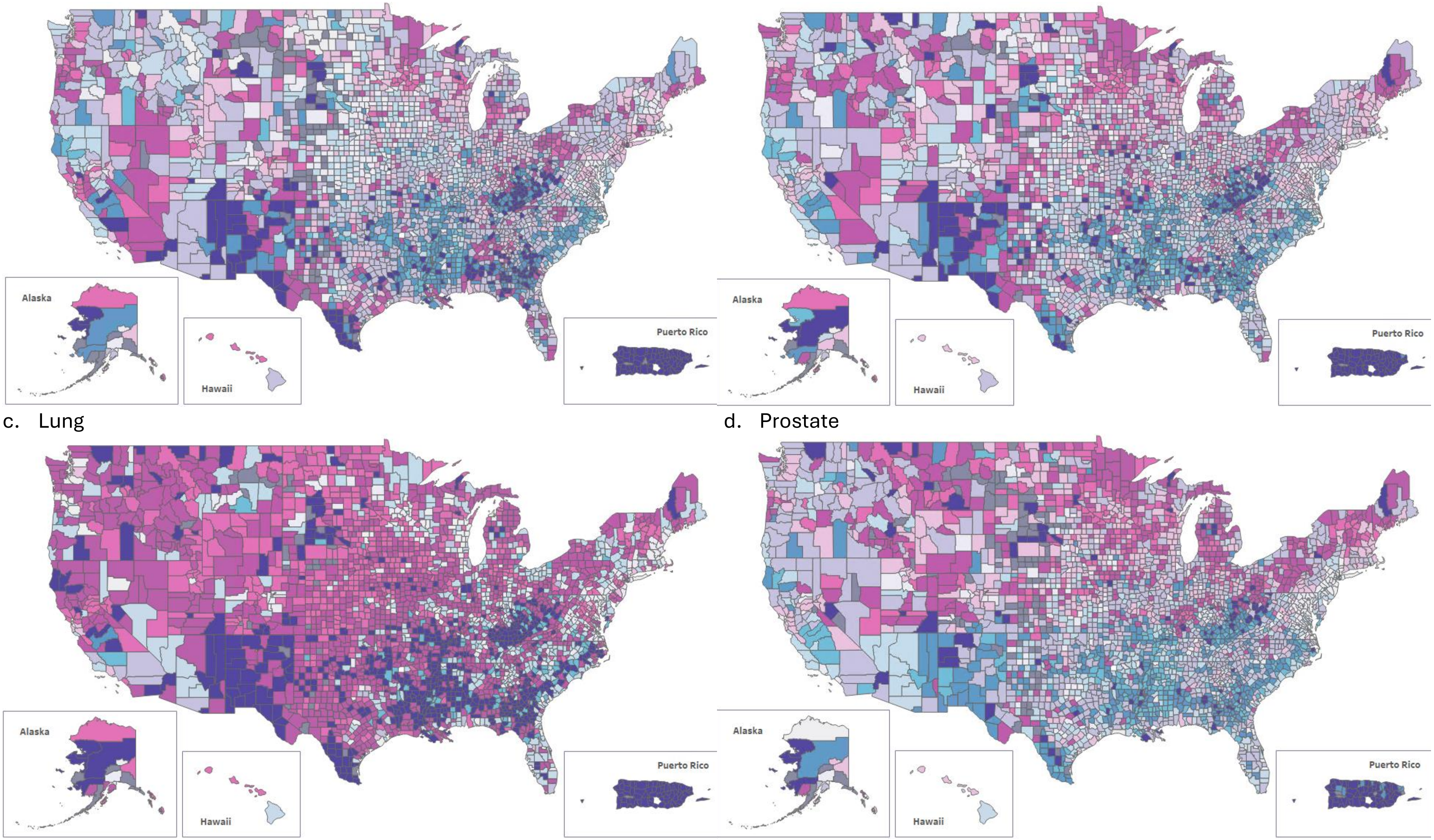

© 2025 Drake AR et al. *JAMA Network Open*.

© 2025 Drake AR et al. *JAMA Network Open*.
