# Supplementary material for: Community Factors and County-Level Cancer Screening, Prevalence, and Mortality
Source: JAMA Netw Open. 2025 Oct 23;8(10):e2537690. doi: 10.1001/jamanetworkopen.2025.37690 (PMC12550642; doi:10.1001/jamanetworkopen.2025.37690)
Supplement: Supplement 2. — Data Sharing Statement [file jamanetwopen-e2537690-s002.pdf]

Data Sharing Statement

Drake. Community Factors and County-Level Cancer Screening, Prevalence, and Mortality. *JAMA Netw Open*. Published October 16, 2025. doi:10.1001/jamanetworkopen.2025.37690

Data
Data available: No

Additional Information
Explanation for why data not available: A majority of our data are Medicare Research Identifiable files that are protected by a DUA.
